# Supplementary material for: The Gasotransmitter Hydrogen Sulfide (H2S) Prevents Pathologic Calcification (PC) in Cartilage
Source: Antioxidants (Basel). 2021 Sep 8;10(9):1433. doi: 10.3390/antiox10091433 (PMC8471338; doi:10.3390/antiox10091433)
Supplement: Supplementary file 1 [file antioxidants-10-01433-s001.zip › antioxidants-1352186-supplementary.pdf]

SUPPLEMENTARY FILES

Supplementary Figure S1: Aged Cse-deficient mice showed exacerbated spontaneous knee PC.

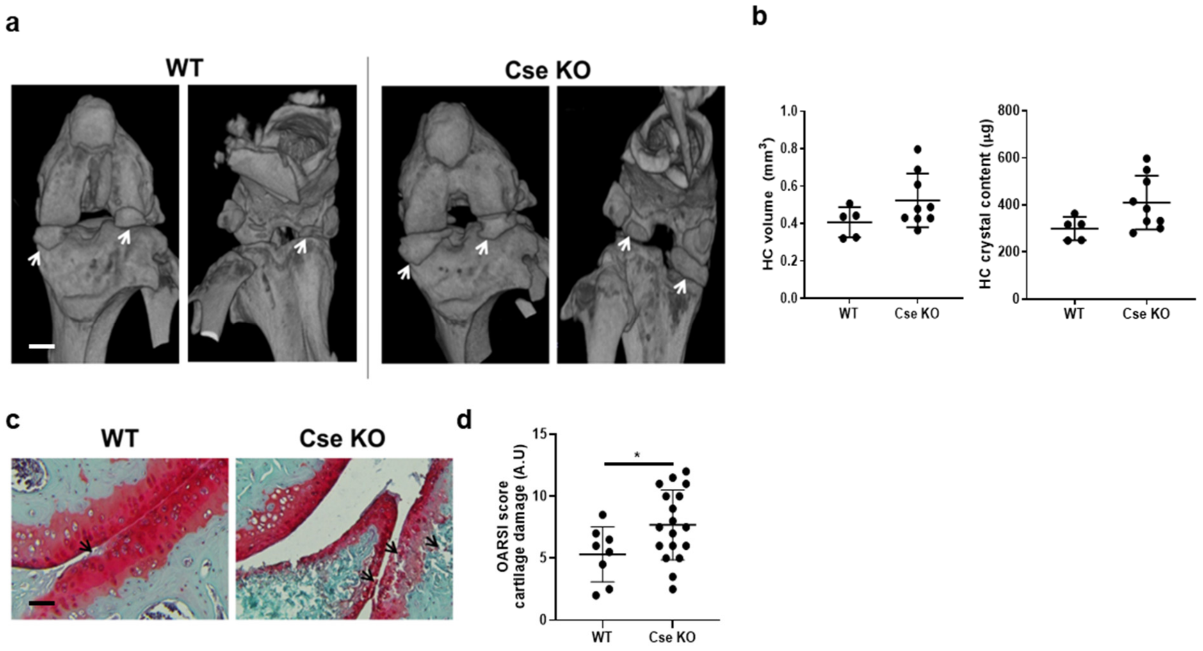

(**a**) Representative micro-CT scan images of WT and Cse KO murine knee joints (anterior and posterior view) from aged mice (61 to 64 weeks old). White lines show: increased size of the anterior portions of menisci in aged WT mice and further exacerbation in Cse KO mice, and PC in the posterior part of the knee, bigger in Cse KO than in WT mice. Scale bars 1mm. (**b**) Quantitative analysis PC volume (mm^3) and the respective crystal content (µg) in WT and Cse KO knees. Knees number WT n=5, Cse KO n=9. (**c**) Representative histologies of WT and Cse KO knees, stained with Safranin-O. Black arrows show increased degenerative OA changes in the articular cartilage of Cse KO mice. Scale bars 150µm. (**d**) Graphs show femoral scoring of cartilage damage and Safranin-O loss, accordingly to OARSI method. Knees number WT n=8, Cse KO n=18. *$p<0.05$.

Supplementary Figure S2: Cse modulation is not associated with altered chondrocyte apoptosis in the surgically-induced model of knee PC

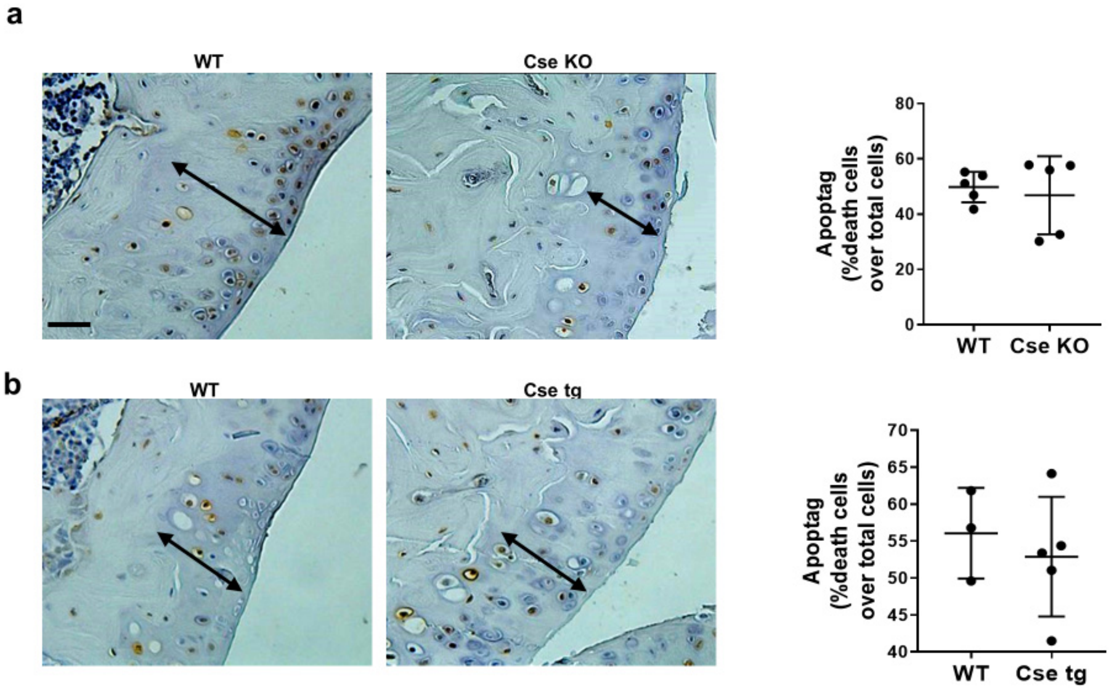

(**a**) Immunohistochemical staining for chondrocyte apoptosis (Apoptag) in knee cartilage from MNX WT and Cse KO mice. One representative staining from 1 out of 5 mice per group is shown. Scale bars 150µm. Mice number WT n=5, Cse KO n=5. The % of Apoptag positive cells over the total amount of cells was counted in three different fields for each mouse. The mean for each mouse is represented in the graphs. (**b**) Immunohistochemical staining for chondrocyte apoptosis (Apoptag) in knee cartilage from WT, Cse tg mice. One representative staining from 1 out of 5 mice per group is shown. Scale bars 150µm. Mice number WT n=3, Cse tg n=5. The % of Apoptag positive cells over the total amount of cells was counted in three different fields for each mouse. The mean for each mouse is represented in the graphs.

Supplementary Figure S3: Set-up of the high-throughput screening assay and identification of a CSE activator compound.

a

$$\text{L-cysteine} + H_2O \xrightarrow{\text{CSE}} \text{Pyruvate} + H_2S + NH_3$$

b

$$\text{Pyruvate} + O_2 + \text{Phosphate} \xrightarrow{\text{POX}} \text{Acetyl phosphate} + CO_2 + H_2O_2$$

$$H_2O_2 + \text{Amplex Red} \xrightarrow{\text{HRP}} \text{Resorufin}$$

c

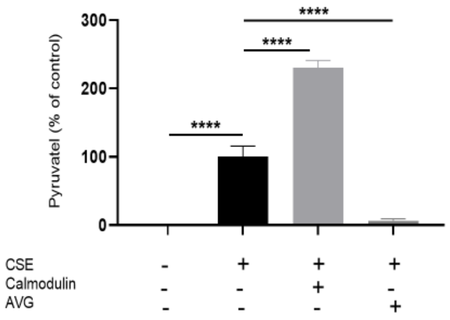

d

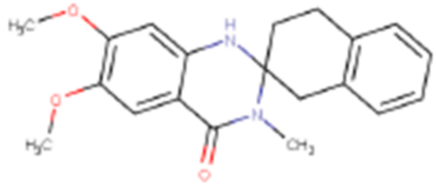

e

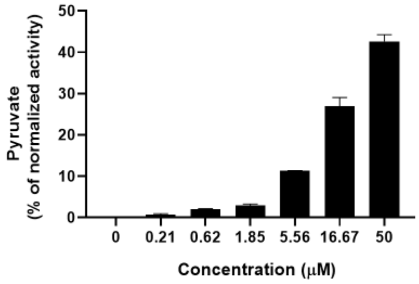

The chemical reactions involved in the 2-step primary screening assay (pyruvate assay) are depicted. In (**a**) recombinant human CSE (hCSE) is incubated with L-cysteine, generating H_2S and pyruvate (68). In (**b**) pyruvate is oxidized by pyruvate oxidase (POX) to generate acetate, carbon dioxide (CO_2) and hydrogen peroxide (H_2O_2). H_2O_2 then reacts with Amplex Red in a 1:1 stoichiometry to form the red fluorescent product, resorufin. (**c**) hCSE-dependent pyruvate measurement without enzyme (no pyruvate production), with the specific CSE inhibitor AVG 5 μM (inhibition of pyruvate production) and with the CSE activator calmodulin 5 μM (increased pyruvate production). Results were expressed as the % of pyruvate generated over hCSE alone. (**d**) Chemical structure of the new small molecule CSE activator SU004. (**e**) CSE-dependent pyruvate upon incubation of hCSE with L-cysteine and with increasing concentration of SU004. The ability of SU004 to activate hCSE was calculated as % of normalized activity.

Supplementary Figure S4: Flow-chart for the high-throughput screening of CSE modulators.

A set of 75000 compounds from a lead-like small molecule library (Exquiron library, Reinach, Switzerland) was used. For screening purpose, the compound stock solutions were transferred from Labcyte 384 LDVplates (LP-0200) to 384 polypropylene plates (Greiner # 781201) using an Echo 555 acoustic liquid handler (LabCyte). The compound solutions (50 nL) were dispensed into columns 1–22 and an equivalent volume of DMSO, and a CSE inhibitor (AVG at 5 μM) used in columns 23 and 24, respectively. Plated compounds were stored at −20°C. The final compound concentration used in the screen was 50 μM, with a final DMSO concentration of 1% in all wells.

For the robotic screening, the primary screening (pyruvate assay, see reactions Supplementary Figure S**3a**,**b**) was miniaturized and optimized on 384-well black polystyrene, flat bottom plates (Greiner). A mix containing 50nM recombinant human CSE (hCSE, ProteoGenix, France), 200 μM L-Cysteine substrate (Sigma) in PBS + 1 mM $CaCl_2$ with 0.1% BSA (Sigma) was dispensed immediately to the assay plates containing compounds (at 50 μM in 1% DMSO final). After 3h at 37°C, pyruvate detection was performed as described, adding 0.25 U/mL pyruvate oxidase (POX, Sorachim), 0.2 U/mL HRP (Sigma P8375), 25μM Ampliflu Red (Sigma 90101) in 100mM potassium phosphate pH 6.7 containing 1mM EDTA, 1 mM $MgCl_2$, 10μM FAD (Sigma F6625), 0.2mM TPP (Sigma C8754). 3 volumes of mix for pyruvate detection were used for 1 volume of mix with hCSE. The plates were incubated for one hour at ambient temperature. The resulting fluorescence signal was measured on EnVision (PerkinElmer) multiplate reader with Excitation 531nm Emission 595nm, with cutoff at 555nm. As a positive control we used hCSE at 500nM final concentration to assess

the assay's quality and plate-to-plate reproducibility. The assay quality was assessed by Z' factor, which was > 0.5 using the following formula: $Z'\text{-factor} = 1-[(3(\sigma p+\sigma n)/(|\mu p-\mu n|)]$, where μn and σn represent the mean and standard deviation of the negative control (50nM CSE), and μp and σp represent the mean and standard deviation of the positive control (500nM CSE). Data were normalized to 0% activation (50nM CSE) and 100% activation (500nM CSE). Activators above 10% of normalized activity were selected and subjected to secondary screens (H_2S assay by the P3 probe and calcification assay by Alizarin Red on cells). Finally, SU004 was selected as hit compound for *in vitro* experiment (Figure **4**).

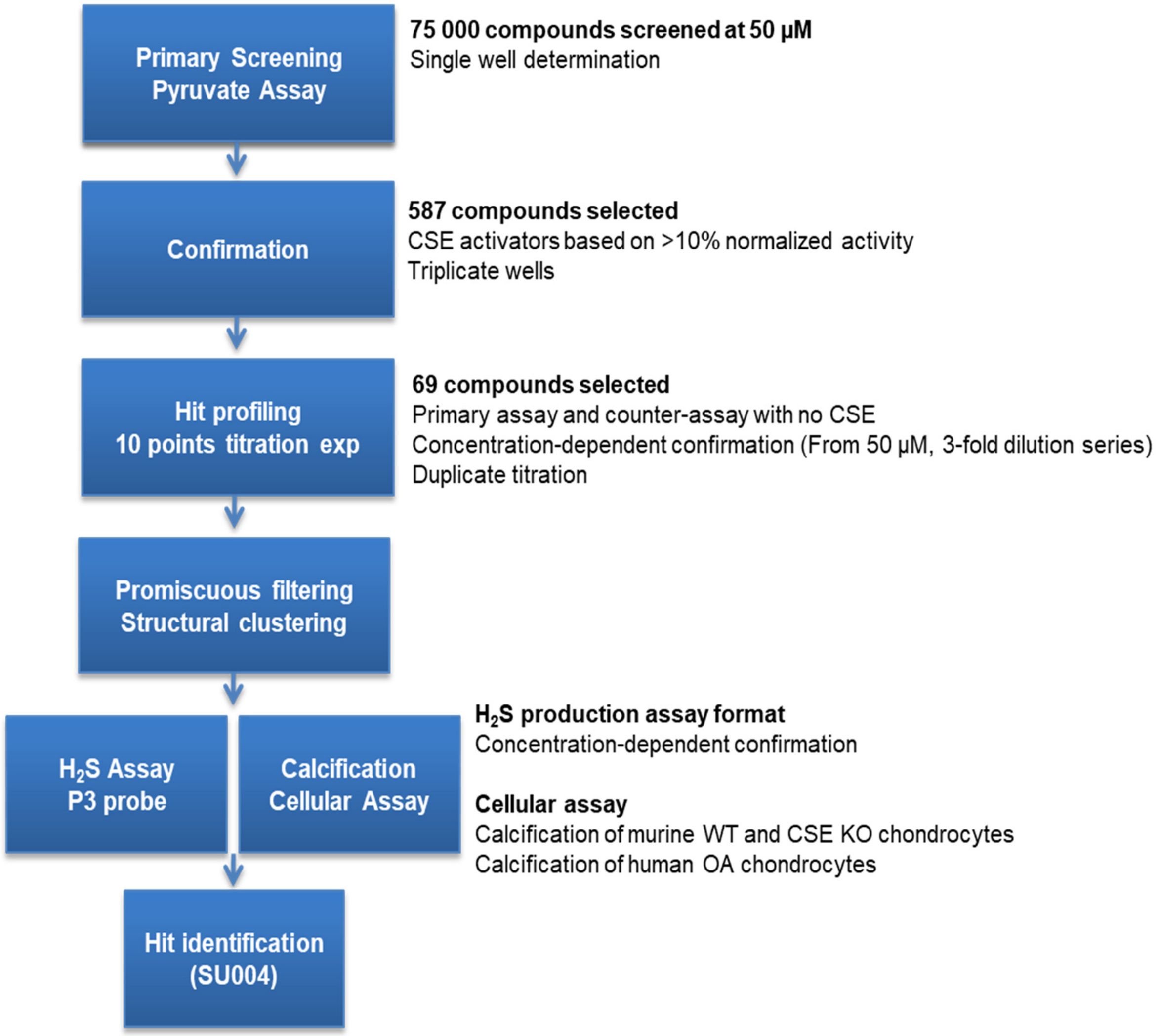

Supplementary Figure S5: CBS is lowly expressed in human and murine cartilage and plays a minor role in chondrocyte mineralization

We firstly assessed CBS expression by immunohistochemistry in both human and murine knee cartilage. In human cartilage, few positive cells for CBS expression were present in superficial cartilage layer, while intermediate and deep layers remained negative (Supplementary Figure S**5a**). In murine healthy cartilage, superficial chondrocytes expressed Cbs. Following induction of experimental PC and OA by meniscectomy Cbs expression was strongly decreased (Supplementary Figure S**5b**). Cbs-deficient chondrocytes (Cbs KO) exhibited the same characteristics of Cse KO cells (i.e increased calcification and increased IL-6 production) (Supplementary Figure S**5c,d**) although the amounts of the formed crystals and of secreted IL-6 were much smaller (9-fold less and 12-fold less respectively compared to the results obtained in Cse KO cells, Figure **2f,g**). Interestingly, Cbs KO chondrocytes showed concomitant increase of both *Cse* and *3-Mst* expression if compared to WT chondrocytes (Supplementary Figure S**5e**). Finally, H_2S levels in Cbs KO chondrocytes were similar to those of the corresponding WT chondrocytes (Figure **5f**) potentially due to compensated production of H_2S by the other two H_2S-producing enzymes.

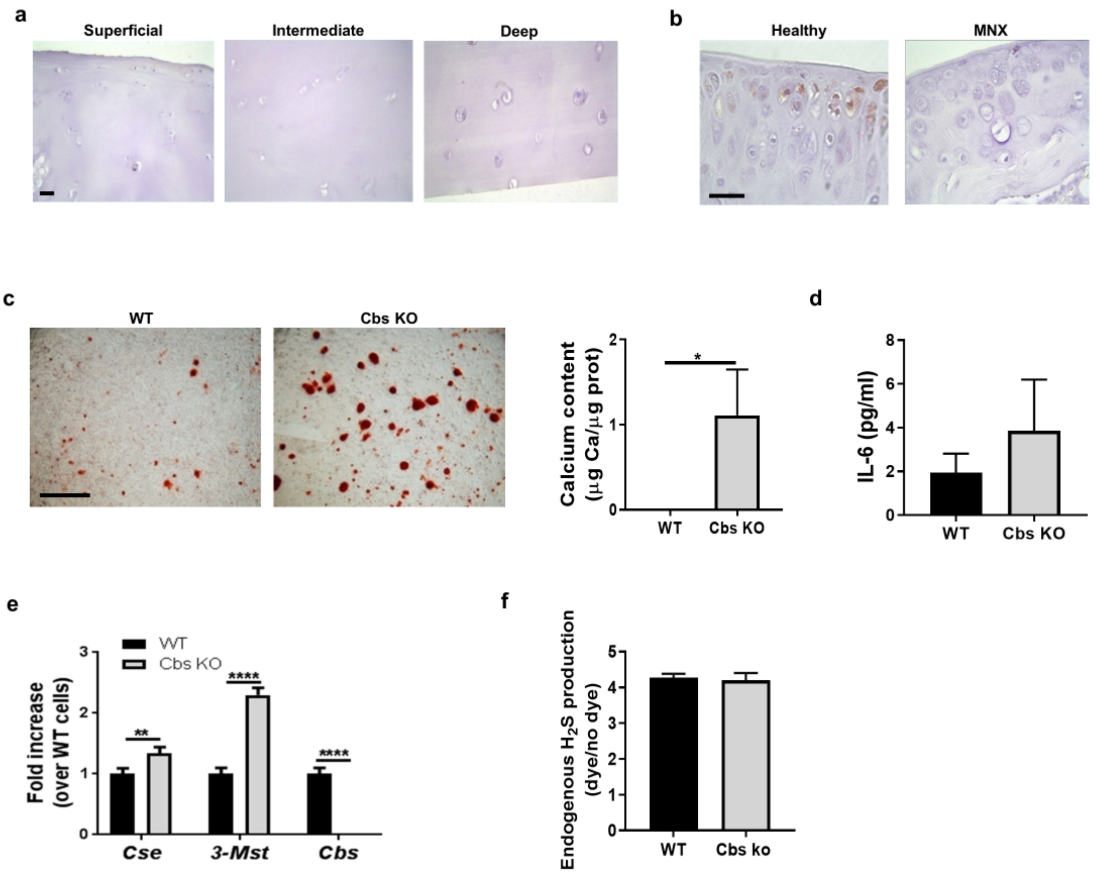

(**a**) CBS immunohistochemical staining in superficial, intermediate and deep layers of knee cartilage from OA patients. One representative staining from 1 out of 5 patients is shown. Scale bars 100µm. (**b**) Cbs immunohistochemical staining in healthy and MNX murine knee cartilage. One representative staining from 1 out of 5 mice is shown. Scale bars 100µm. (**c**) Alizarin red staining of WT and Cbs KO chondrocytes cultured for 14 days in calcification medium. Pictures represent triplicates from one experiment of three independent experiments. The graph represents calcium content in the cell monolayer, expressed as % over Nt cells, at 14 days of culture. (**d**) IL-6 secretion by WT and Cbs KO chondrocytes cultured for 14 days in calcification medium. (**e**) qRT-PCR of the indicated genes in WT and Cbs KO chondrocytes. (**f**) FACS analysis of endogenous H_2S production by WT, and Cbs KO chondrocytes by the P3 probe.
